# Characterization of Self-Growing Biomaterials Made of Fungal Mycelium and Various Lignocellulose-Containing Ingredients

**DOI:** 10.3390/ma15217608

**Published:** 2022-10-29

**Authors:** Ilze Irbe, Gustavs Daniels Loris, Inese Filipova, Laura Andze, Marite Skute

**Affiliations:** 1 Latvian State Institute of Wood Chemistry, Dzerbenes iela 27, LV 1006 Riga, Latvia; 2 Faculty of Biology, University of Latvia, Raina bulvaris 19, LV 1586 Riga, Latvia

**Keywords:** biodegradability, birch bark, birch sawdust, compression, hemp shives, lignocellulose, mold resistance, mycelium biocomposites, water absorption, wheat bran

## Abstract

In this study, novel blends of mycelium biocomposites (MB) were developed. Various combinations of birch sawdust and hemp shives with birch bark (BB) and wheat bran (WB) additives were inoculated with basidiomycete *Trametes versicolor* to produce self-growing biomaterials. MB were characterized according to mycelial biomass increment in final samples, changes in chemical composition, elemental (C, H, N) analyses, granulometry of substrates, water-related and mechanical properties, as well as mold resistance and biodegradability. The mycelial biomass in manufactured MB increased by ~100% and ~50% in hemp and sawdust substrates, respectively. The lignocellulose ingredients during fungal growth were degraded as follows: cellulose up to 7% and 28% in sawdust and hemp substrates, respectively, and lignin in the range of 13% in both substrates. A larger granulometric fraction in hemp MB ensured higher strength property but weakened water absorption (600–880%) performance. Perspective MB combinations regarding strength performance were hemp/BB and pure hemp MB (σ10 0.19–0.20 MPa; E 2.9 MPa), as well as sawdust/WB combination (σ10 0.23 MPa; E 2.9 MPa). WB positively affected fungal biomass yield, but elevated water absorption ability. WB improved compressive strength in the sawdust samples but decreased it in the hemp samples. BB supplement reduced water absorption by more than 100% and increased the density of sawdust and hemp samples. All MB samples were susceptible to mold contamination after full water immersion, with identified fungal genera *Rhizopus*, *Trichoderma* and *Achremonium*. The MB exhibited high biodegradability after 12 weeks’ exposure in compost, and are therefore competitive with non-biodegradable synthetic foam materials.

## 1. Introduction

The amount of plastics accumulating in the environment is growing rapidly, yet our understanding of its persistence is very limited. Plastic waste is currently generated at a rate approaching 400 Mt year^−1^ [1]. Most building and packaging materials are non-biodegradable and become waste after their use. In some cases, the waste is burned, contributing to air pollution and creating a public health hazard [2]. There is an urgent need for new ecological materials that will biodegrade over time after their use in contrast to petroleum-based products which decompose in hundreds or thousands of years. The development of natural biocomposites in compliance with eco-design principles is a significant contribution towards the circular bioeconomy.

Existing agricultural and wood processing industries produce lignocellulose by-products, which often are not managed in a sustainable way and are treated as waste. There is an option to utilize this waste by incorporation in composite materials. Mycelium biocomposites (MB) are a type of biocomposites relying on the valorization of lignocellulosic wastes and the natural growth of the fungal organism. While growing, the fungus reinforces the substrate, which is partially replaced by the biomass of the fungus itself, resulting in a biocomposite material [3]. Lignocellulose is a structural component of wood and non-wood fibers and represents a major source of renewable organic matter. Cellulose, hemicelluloses, and pectins are the major polymeric carbohydrates, composing about 75% of the wood cell wall. Lignin, an aromatic heteropolymer, makes up most of the remaining cell wall substance. The principal source of nutrition for most wood-inhabiting microorganisms is the carbohydrates present in cell walls and storage tissues [4]. White-rot fungi are the most efficient and extensive lignocellulose degraders due to their capability of producing a variety of hydrolytic and oxidative enzymes acting with various specificities and synergies [5]. They belong to basidiomycetes and are unique in that they can efficiently mineralize lignin from wood in addition to the wood polysaccharides to CO_2_ and H_2_O [6]. In their turn, the fungal cell walls consist of 80 to 90% polysaccharides, with the remainder composed of proteins and lipids. Chitin is the principal skeletal material and is present in the inner wall of most septate fungi. The chitinous nature of the cell wall is one of the fundamental differences separating most fungi from plants [4].

*Trametes versicolor* (L.) Lloyd is one of the fungal species used in the manufacturing of MB. It belongs to the phylum Basidiomycota and is a common species found throughout Europe. The fungus causes white rot mainly in hardwoods [7]. *T. versicolor* has been employed also in traditional medicine practises and studied for use in cancer therapy [8], laccase production for biotechnological and industrial applications [9], described for antiviral, anti-inflammatory, antimalarial activity, diabetic, and hepatitis treatment [10] and novel biopolymer production from the fungal hyphae and cellulose fibres [11].

A unique feature of MB is the wide diversity of technical and aesthetic properties. The final MB products can be shaped to produce acoustic absorption panels [12], insulating panels and packaging materials [3], panelling and flooring, bricks or new-design objects [13]. MB production involves low embodied energy, the resulting materials are biodegradable, and they have a potential for cost-effectiveness [14]. Due to their low thermal conductivity, high acoustic absorption and fire safety properties outperforming traditional construction materials, such as synthetic foams and engineered woods, MB show particular promise as thermal and acoustic insulation foams [15,16]. The properties of mycelium-based foams surpassed by expanded polystyrene foams are density, flexural strength, and water absorbance. Thus, to meet the general performance requirements of commercial synthetic foams, investigation needs to be focused on reducing material density, increasing flexural strength, and decreasing water permeability [17]. Currently, the MB are commercially developed by *Ecovative Design* (packaging and construction materials), *Grown.Bio* (packaging and interior design products) and *Mogu* (acoustic and flooring panels).

Since MB development is still an innovative field, the evaluation of important technological properties and standardization is still in progress. MB can display an enormous variability on the basis of the fungal species and strains, substrate composition and structure, incubation conditions and duration [13,18]. The available literature and knowledge on MB development are currently very fragmented. There are no standardized and comparable methods of the production parameters and mechanical properties of the material. The different process parameters from the fungal morphology and feedstock type to processing conditions and mechanical properties is a challenging task to interpret, evaluate and compare the results of different studies [19]. The increased number of studies on mycelium materials will certainly improve the knowledge for the development of a new niche of ecological products. In this context, our study brings additional knowledge on specific MB combinations and their properties. Hemp shives, birch sawdust and birch bark are by-products that require more efficient utilization and alternative application to maximize their potential. We investigated these wood and non-wood residual materials in different combinations to manufacture novel MB materials. Besides determination of mechanical and biodegradation properties, the novelty was brought with (i) analyses of chemical composition (cellulose, lignin) and elements (C, H, N) in MB samples, (ii) fungal biomass increment in MB samples, and (iii) mold susceptibility.

## 2. Materials and Methods

The lignocellulose substrates for development of MB were obtained from the local industry. Hemp (*Cannabis sativa* L.) shives (HS) were collected from the agricultural waste stream and silver birch (*Betula pendula* Roth) sawdust (BS) and birch bark (BB) from the wood processing industry. Three combinations of hemp and sawdust containing substrates were made with the additives wheat bran (WB) (8% wt from substrate), BB (30% wt from substrate) and distilled water (75% wt from substrate):

Variant I-substrate (hemp or sawdust), WB, distilled water

Variant II-substrate (hemp or sawdust), WB, BB, distilled water

Variant III-substrate (hemp or sawdust), distilled water.

The particle size and percentage of fractions in the hemp and sawdust substrates were determined by sieving, vertically combining and shaking sieves of four different pore sizes, namely, 10 mm, 7 mm, 5 mm and 3 mm. Each granulometric fraction remaining on the sieve was weighed and the percentage of the total weight was calculated (Table 1). The particle size of BB was ≤5 mm.

The mycelium of white-rot fungus *Trametes versicolor* CTB 863 A was cultivated in Petri dishes on a medium containing 5% malt extract concentrate and 2% agar (Sigma-Aldrich, St. Louis, MI, USA), pH 6, at 22 °C and 70% relative humidity (RH). Fungal inoculum of 1 cm^2^ size in sterile conditions was transferred to 250 mL flasks filled with 100 mL of the standard medium, containing glucose (15.0 g L^−1^), peptone (3.0 g L^−1^), yeast extract (3.0 g L^−1^), NaH_2_PO_4_ (0.8 g L^−1^), K_2_HPO_4_ (0.4 g L^−1^), MgSO_4_ (0.5 g L^−1^), pH 6.3, and cultivated in a rotary shaker Multitron (Infors HT, Switzerland) at 150 rpm and 27 °C. After 14 days of cultivation, mycelial pellets were homogenized using a Waring laboratory blender and mixed with sterile substrate variants in 1 L glass jars. The ratio of liquid inoculum to substrate was 1:1.

After 14 days of growth, the mycelium-substrate biomass was placed in plastic molds to continue growth for a further seven days. The developed MB samples with size 150 × 50 × 50 mm were withdrawn from the molds and dried in an oven at 70 °C for 24 h.

The moisture content (%) before and after development of MB was calculated from the wet weight and final dry weight. The conditioned MB samples were cut in specimens with dimensions of 30 × 30 × 30 mm for further analyses.

The changes in mycelial biomass during MB development were characterized by the determination of initial inoculated mycelium (%) and the final mycelium amount (%) in developed MB samples. The initial biomass amount in the liquid medium was determined after 14 days of submerged cultivation by filtering, washing, and drying in an oven at 70 °C until constant weight. The dry biomass concentration was expressed in grams per 100 mL of medium. The mycelial biomass in final MB samples was determined by grinding of MB samples with an electric universal mill M20 (IKA-Werke, Germany) for 2 × 20 s. An analytical sieve shaker AS200 (Retsch, Germany) with 1 mm sieve mesh was used for the separation of fractions. The obtained substrate and mycelium fractions were collected separately and weighed. After weighing, the sieved fractions were analyzed with a stereomicroscope S9i (Leica, Germany) to determine the degree of impurities.

The lignocellulose and ash content (i) in the raw materials, (ii) in the middle phase of MB development (14 days), and (iii) in the final MB product (21 days) were analyzed by chemical methods. MB samples were ground in an electric mill for 35–40 s and sequentially sieved with a vibrating sieve shaker to remove mycelium and select the appropriate particle size for chemical analysis.

Cellulose was determined by the Kürschner-Hoffer method and lignin by the Klason method [20]. Ash was determined according to TAPPI T 210:2003. The content of hemicelluloses was determined empirically as the remaining portion after analyses of lignocellulose and ash content.

For elemental analysis (C, H, N) according to CEN/TS 15104:2011, homogenized samples (30 mg) were packed in a tin foil, weighed, placed into the carousel of an automatic specimen feeder and analyzed with Vario MACRO CHNS (Elementar Analysensysteme GmbH, Germany) with a combustion tube temperature of 1150 °C. The original matrix of the specimen was destroyed under these conditions through subsequent catalytic reactions.

The water absorption and volumetric swelling were measured according to the ASTM D1037: 2012. Six conditioned specimens from each MB variant were selected and immersed in distilled water for 2 h and 24 h. The water absorption and volumetric swelling values were determined from the weight and volume difference in relation to the initial weight and volume.

The compression test was performed on the basis of the European standard ISO 844: 2009. Two mechanical parameters were determined: compressive strength and elastic modulus (E). The Zwick/Roell Z010 universal testing machine and the original Zwick/Roell software were used. Six conditioned specimens from each MB variant were weighed and their density was determined. The mechanical load at 10% relative deformation of the specimen was determined. The compression speed corresponded to 10% def/min, and the preload was 2 N. During the test, a force-displacement graph was recorded, from which the elastic modulus (E) was determined by drawing a tangent to the steepest part of the curve.

After 24 h of water absorption test, the MB specimens were directly transferred to the glass containers and subjected to the natural mold contamination from the indoor air. The containers were partly covered with a glass lid, allowing air circulation from the indoor environment. The experiment was performed in a cultivation chamber at 22 °C and 70% RH for 14 days. Fungal growth was periodically evaluated according to the rating scale from 0 to 4, where 0—clean, 0% attack; 1—trace, 5% growth; 2—slight, 6–25% growth; 3—medium, 26–50% growth; 4—severe, >50% growth.

For mold identification, the colonies were slightly touched by an adhesive tape, after which the tape was transferred to a microscope slide with a drop of mounting medium (*lactophenol blue* solution). The fungal samples were investigated with a light microscope DMLB (Leica, Germany) at magnifications of ×50, ×100, ×200 and ×400.

A biodegradation test was carried out according to modified EN 14045:2003 in a climatic chamber at 20 °C and 65% RH. Three specimens from each variant were subjected to biodegradation into ecologically clean, natural compost produced by Zeltabele Ltd. (Latvia). Each specimen was weighted and placed in 0.5 L individual box with compost, and these boxes were inserted in a compost container with volume of 130 L. The total test duration was 12 weeks. Within this period the specimens were inspected every week for the first 4 weeks and every second week for the remaining duration. The substrate temperature and moisture content were measured regularly. After 12 weeks the individual specimen boxes were withdrawn from the container, dried in oven at 40 °C for 3 days and sieved using 2 mm sieve to separate non-degraded particles.

The statistical analyses were carried with RStudio (v.1.3.1093). For group comparisons the parametric ANOVA (f:aov) with Tukey-Kramer (f:Tukey HSD) post-hoc tests was used. Correlations between parameters were carried out by Pearson correlation (f:cor.mtest). All statistical tests were done at the significance level α = 0.05 (*p* ≤ 0.05).

## 3. Results and Discussion

### 3.1. Mycelial Biomass

The average moisture content before MB development was 65.8% for hemp and 67.7% for sawdust samples. In finalized MB the average moisture was 52.8% and 49.3% for hemp and sawdust samples, respectively. As the MB result from a biological process, adequate moisture in the substrate is needed to allow fungus to grow and carry out its physiological functions. This parameter is strongly related to the composition of the substrate and the fungal species under examination. Moisture of at least 60% is reported for the development of mycelium materials [21].

Table 2 shows the increment of fungal mycelium during MB development in 21 days from the inoculation to the final stage. Three hemp substrate variants caused around 100% increment of mycelium, giving evidence of easily available substrate for fungal growth. It is significant that hemp III variant showed a little lower rate of hyphal growth, probably due to the absence of WB as a nutrition supplement. In their turn, sawdust variants I and II had mycelium increments of about 50%, that is half less than equal combinations of hemp substrates. Sawdust variant III without added WB supplement showed only a 10% increase in mycelial biomass. Hemp was a more accessible substrate to the enzymatic action than sawdust because of the different ratio of lignocellulose content, and higher mineral and nitrogen content as nutrition (Section 3.2 and Section 3.3). It is noteworthy that this was our first attempt to characterise the relation between fungus and substrate during the growth phase. Consequently, some deviations in measurements after separation of mycelial and substrate fractions because of impurities cannot be excluded. Mycelium separation methodology will be optimized in future studies.

### 3.2. Chemical Analyses of MB Components

The chemical composition of MB ingredients is given in Table 3.

BS contained a higher amount of cellulose (48%) than HS (45%). Lignin content in both substrates was almost equal, i.e., 23–24%. The highest total phenolic content, including lignin, suberin, and extractives, was determined in BB (79%). The BB contained up to 25.7% of biologically active compounds (betulin, lupeol, betulinic acid) in outer bark [22], and high amounts of phenolic compounds, among others, in inner bark [23]. Suberin compounds from BB are reported for their hydrophobic properties [24]. The addition of BB to our MB sample variant II was aimed at potentially improving the mechanical and water absorption properties by increasing the material density and hydrophobic performance, respectively.

A low percentage of cellulose and lignin in WB (Table 3) was related to a high content of other components, including hemicelluloses, starch, protein, and fat. According to the literature [25], the fibre content in WB varies from 40 to 53% of the dry matter, starch from 9 to 25%, protein around 16% and fat around 5%. 22–30% of the dietary fibre was arabinoxylan, and the remaining was cellulose (9–12%), lignin (3–5%), fructan (3–4%) and mixed linked β-glucan (2.2–2.6%). WB is known as a cheap and widely used by-product, and a well-suited growth substrate promoting the lignocellulolytic enzyme production by the majority of basidiomycetes [26,27].

Ash content is a measure of the mineral content and other inorganic matter in biomass. The highest ash content was determined in WB. This is in line with another study, in which ash content was reported around at 5.5–6.5% [25]. Ash content of different biomass can vary significantly. For example, herbaceous biomass have higher ash content than wood because they uptake more nutrients during growth [28].

**Figure 1:**
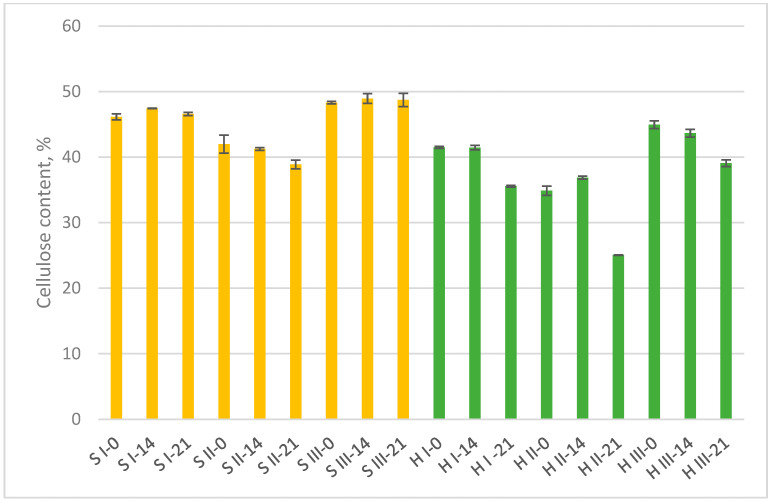
Cellulose content at three mycelium biocomposites’ development stages: start point (0 days), 14 days, and 21 days (final sample). S = sawdust; H = hemp; I, II, III = variants.

The changes in chemical composition of the MB ingredients in two development stages (after 14 and 21 day) were analyzed to understand the level of substrate decomposition during fungal growth. Cellulose, hemicelluloses, and lignin are complex polymers that must be reduced by enzymes to small diffusible units before they can be absorbed and utilized by fungal cells. This process of external digestion begins with the release of exoenzymes into the substrate and ends when the soluble products of digestion diffuse back to the hyphal wall. These polymer residues of the decay process are primarily simple sugars (glucose, xylose, galactose, etc.), which readily pass through the fungal cell wall [4]. The data on changes in cellulose content during fungal growth and biodegradation of MB substrates are summarised in Figure 1. The highest cellulose content within the sawdust and hemp substrate groups was observed in variant III (without additives). This indicated that cellulose was less degraded in this variant. Sawdust I samples during fungal growth within 21 days showed minor changes in cellulose content. It would be due to the primary consumption of WB with high content of hemicelluloses, starch, protein and minerals. Sawdust II had a relatively lower cellulose content in all development stages because of added bark with higher lignin proportion. Sawdust II also contained WB that could promote mycelium growth. The highest cellulose degradation in sawdust group demonstrated S II with a 7% decrease in 21 days. Hemp samples showed more distinct decrease in cellulose content in all variants. Hemp cellulose was degraded in range of 13–28%, depending on the variant, with the highest decrease in hemp II variant.

Bark additive neither in sawdust II nor hemp II variant limited cellulose degradation. The antimicrobial activity of white birch (*Betula papyrifera*) bark extract was reported by Blondeau et al. (2020) [29]. Silver birch bark was reported for its content of many biologically active substances with antioxidative mechanisms that are used in medicine and cosmetics [30]. These studies have described a specific application of bark extractives depending on the active substances. In our study, the bark fraction, integrated in MB materials, did not reduce cellulose breakdown.

**Figure 2:**
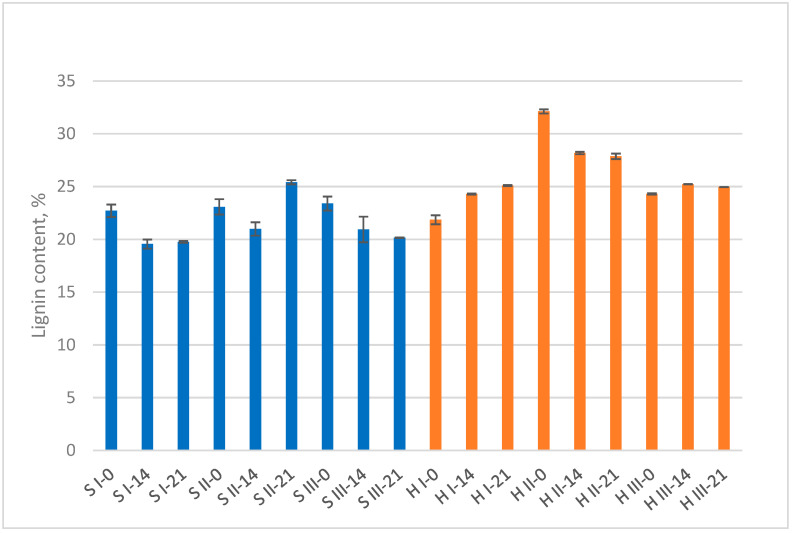
Lignin content at three mycelium biocomposites’ development stages: start point (0 days), 14 days, and 21 days (final sample). S = sawdust; H = hemp; I, II, III = variants.

The highest lignin content within sawdust and hemp groups was determined in bark containing variant II (Figure 2). Sawdust variants I and III showed a reduction in lignin by 13–14% from the initial content. In S II variant, lignin also had a tendency to decline with the exception of the final development stage when it had relatively increased on the basis of some cellulose degradation (Figure 1). Hemp samples with more easily available cellulose source for enzymatic action showed a relative increase in lignin content (variant I and III) on the basis of cellulose degradation. The hemp II showed lignin reduction by 13%. Bark additive did not suppress cellulose and lignin enzymatic degradation both in sawdust and hemp samples.

The MB samples are complex media where each ingredient and its proportion can affect fungal growth and substrate degradation. WB as an additional supplement was the primary source of nutrition that resulted in mycelial biomass increment (Table 2) especially in the case of sawdust variants I and II. The WB has been shown to promote the production of hydrolytic and ligninolytic enzymes for lignocellulose degradation [31]. In pure S III variant where sawdust was the only lignocellulose source, the fungal biomass increased negligibly. Lignin degradation in S III variant was detected, but cellulose content was not. The duration of MB development was probably too short for the fungus to spread extensively within the sawdust and degrade cellulose in measurable amounts. In lignified wood cells, the cellulose is generally surrounded by the hemicelluloses and lignin matrix. White-rot fungi vary considerably in the relative rates at which they attack lignin and carbohydrates in woody tissues. *T. versicolor* colonize cell lumina and cause cell wall erosion referred as nonselective or simultaneous rot. However, the ratio by which lignin, hemicellulose and cellulose are decayed by a selected fungus can differ enormously, and even different strains of the same species may behave differently on the same kind of wood [32]. Thus, the chemical analyses of MB samples shows the general tendency of substrate degradation during the formation of composite material.

### 3.3. Ash Content and Elemental Composition

The ash content characterises the inorganic matter in the material. Results showed that the ash content depended on the MB development stage, substrate type and ingredients of each variant (Table 4). The lowest amount was determined at the initial stage of MB development with pure ingredients. In the next development stages (14 and 21 day) the ash content gradually increased and reached the highest amount in final MB samples. The ash in the sawdust group ranged from 0.4 to 1.9% and in the hemp group from 1.7 to 4.4%. The increase in ash content during MB development can be explained by substrate degradation which led to the rise of relative ash content. This feature was observed in all MB variants. The ash increased 1.5–2 times from the initial to the final MB development stage.

The elements N, C, and H in the MB samples were associated with both the pure substrate components and the fungal hyphae. The trace amount of nitrogen was found in pure substrates in sawdust variants from 0.25 to 0.34%, and in hemp variants from 0.60 to 0.74%. The nitrogen increased after 14 and 21 days of fungal growth in MB samples, with the exception of the S III variant, which is in line with poor mycelial growth (Table 2). Nitrogen is structurally and functionally linked with fungal cellular functions as organic amino nitrogen in proteins and enzymes [33]. Nitrogen in a higher amount was detected in hemp MB, supporting the finding about noticeable mycelium increment in hemp samples (Table 2). Carbon ranged from 47% to 54% in sawdust samples, with a higher amount in S II variant with bark additive. Similar carbon amount was detected in hemp samples, i.e., 46–50% depending on the MB variant. Hydrogen amounts in all MB variants were similar, within the range of 5–6%. Amounts of carbon and hydrogen are basically determined by the substrates and are close to the previous findings [34,35]. Carbon is also a structural element of fungal cells (chitin, glucans, mannans and glycoproteins) in combination with hydrogen, oxygen, and nitrogen [33].

### 3.4. Water-Related Properties

**Figure 3:**
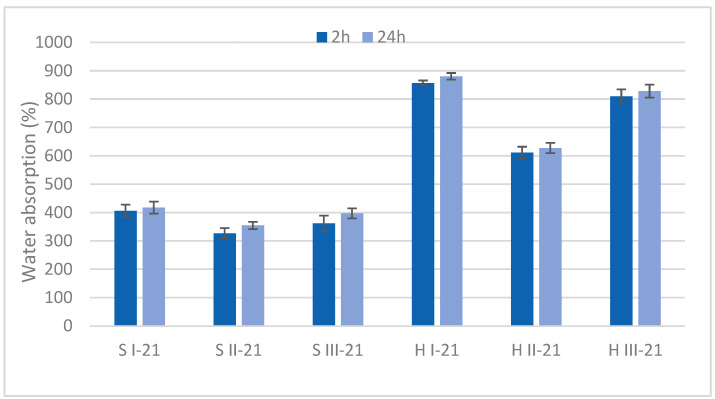
Water absorption of final mycelium biocomposites after 21-day development. S = sawdust; H = hemp; I, II, III = variants.

The water absorption properties of developed MB specimens were determined after 2 h and 24 h immersion (Figure 3). Absorption within 2 h was a critical duration after which the water uptake of MB was limited. Hemp specimens had the highest water absorption, reaching 630–880% after 24 h. Significant difference (*p* < 0.05) was determined in all hemp variants both after 2 h and 24 h, with the highest absorption for variant H I, followed by H III, and H II with the lowest value.

Sawdust specimens showed half the absorption showed by hemp, i.e., in the 350–400% range. Significant differences after 2 h was found among all sawdust variants, and after 24 h variant S II significantly differed from variants S I and S III. Significant correlation was observed between 2 h and 24 h absorption (r = 0.96).

In both substrate groups significant differences were observed after 2 h absorption among all variants, with the exception of sawdust III from S I and S II. The difference was significantly higher in the hemp group which was more affected by the variant.

A lower absorption within sawdust and hemp groups in variant II proved the effect of hydrophobic behavior of bark component in samples, reducing absorption by more than 100%. However, the absorption level of bark containing MB was considerably higher than for other reported composite materials. For example, water absorption after 24 h for particleboards manufactured from eucalyptus wood was 43%, and for rice husk and bamboo particles 67–72% [36]. A high water absorption capacity of MB has to be significantly reduced if the material is foreseen for building materials. However, this MB property can be successfully used in other applications such as biosorption.

**Figure 4:**
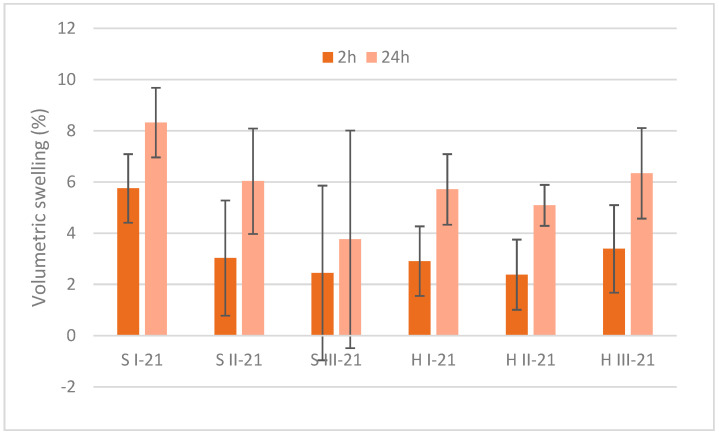
Volumetric swelling of final mycelium biocomposites after 21 day development. S = sawdust; H = hemp; I, II, III = variants.

The volumetric swelling of MB specimens after 2 h and 24 h is shown in Figure 4. Significant positive correlation in sawdust variants after 2 h and 24 h was determined (r = 0.65). The highest swelling was observed for sawdust S I in 2 h and 24 h period, ~6% and 8%, respectively. The swelling of other sawdust and hemp specimens after 2 h was in 2–3% range, and after 24 h it was around 6%. Contrary to the water absorption properties, the difference in volumetric swelling between 2 h and 24 h in most specimens was twice higher. There was no clear effect of bark additive in the reduction of swelling performance.

The volumetric swelling of MB specimens was lower than reported for other composite materials. The swelling of rapeseed particles reached ~35%, but for particleboards and OSB ~20% [37]. A low volumetric swelling versus high water absorption can be explained by the porous structure of MB materials that were filled with high amounts of liquid with no further swelling capacity.

The water related properties of MB specimens depended on the properties of composite ingredients and the density of samples (Section 3.5). Significant, negative correlation between density and absorption in sawdust samples was observed after 24 h (r= −0.59) and positive correlation between density and swelling (r = 0.65). Strong, negative and significant correlation was found between hemp density and water absorption after 2 h and 24 h (r= −1.0 and r= −0.99, respectively.)

Cell wall microstructure, porosity and absolute density are of high importance for the characterization of bio-aggregated materials. The average accessible porosity of HS is reported as 76.6% [38]. In the study of Hussain et al. (2018) [39], pure HS had water absorption with a 400% increase in its original mass. This was mainly caused by its highly porous structure and tendency to absorb water due to its hydrophilic nature. Stevulova et al. (2015) [40] reported that water sorption behavior of hemp composites depended on mean particle length and on binder nature. A longer mean particle length acquired higher values of water content. The difference in particle size was pronounced also in the MB specimens. In hemp specimens, the longer particle fractions dominated the smaller ones (Table 1) that led to a higher water absorption. The dominance of smaller particles in sawdust specimens made a denser material with a lower water uptake. Differences in particle size proportion in hemp and sawdust samples obviously affected the volumetric swelling. The hemp specimens with lower density (Table 5) and longer particles had bigger voids causing a higher water uptake and lower swelling.

The role of mycelium in water absorption is disputable. A low water uptake in mycelium is consistent with the hydrophobic nature of some fungal proteins and glycol-proteins, such as hydrophobins [18]. Although the comprehensive biomass provided by hydrophobins is negligible, these proteins represent an important driver in the physical properties of mycelia [41]. However, a higher fungal biomass concentration in the hemp specimens (Table 2) did not limit water absorption. Obviously, water absorption behavior in MB specimens was determined by the substrate rather than by its mycelium properties.

### 3.5. Mechanical Properties

The density of sawdust specimens was twice that of the hemp group (Table 5). The highest density within each group was determined for variant II with bark additive. The sawdust group with dominant smallest fraction < 3 mm (Table 1) had increased density. Besides, the birch wood density was ten times higher (0.650 g cm^−3^) than that of hemp (0.068 g cm^−3^). The influence of particle size on density more feasibly could have been evaluated within the group of the same substrate. The density significantly differed (*p* < 0.05) in all variants within the sawdust and hemp groups.

The highest compressive strength in all MB specimens was detected in S I variant (Table 5). The samples of S III variant showed significantly lower strength performance and elastic modulus (*p* < 0.05) because of limited mycelial growth and particle bonding. In the sawdust group, positive correlation between strength and elastic modulus (*p* < 0.05; r = 0.99), as well as between density and strength (r = 0.73) and modulus (r = 0.69).

In the hemp group, the highest strength was 0.198 MPa in H II variant. Variant H I significantly differed from H II and H III, with the latter having the lowest strength. Significant positive correlation in hemp variants was between strength and modulus (r = 0.9) and between density and strength (r = 0.59). The effect of bark additive on strength performance in hemp group was evident.

If all MB variants are compared, those combinations regarding strength performance had hemp II with bark (0.20 MPa), hemp III without additives (0.19 MPa), and sawdust S I with bran additive (0.22 MPa). These variants provided also the highest elastic modulus. The reviewed compressive strength values of mycelium composites from other studies were in the range 0.001–0.072 MPa and 0.35–0.57 MPa [17]. The main competitor of MB is expanded polystyrene (EPS) with lower density (0.012–0.048 g cm^−1^) and higher compressive strength (0.035–0.69 MPa) [17]. These EPS values show that our MB materials are competitive with some commercial EPS types.

Larger granulometric fractions in hemp specimens ensured high strength properties but weakened the performance of water absorption. These results are in line with another study where the effect of wood particle size on mechanical and water-related properties showed that the large-sized wood particles in wood plastic composites provided better mechanical properties than did the small size. However, larger particles had lower quality water resistance properties: water absorption and thickness swelling were greater by about 20% [42].

### 3.6. Susceptibility to Mold Growth

**Figure 5:**
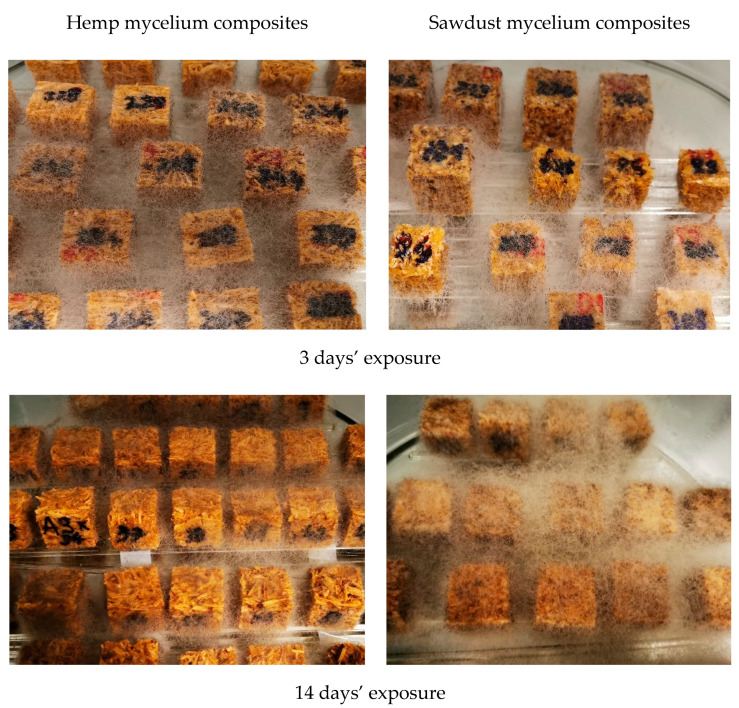
Hemp and sawdust containing mycelium biocomposites after 3 and 14 days exposure to mold contamination.

The mold growth on all hemp and sawdust substrate variants was observed already after 3 days exposure, showing that up to 50% (grade 3) and >50% (grade 4) of the MB surface was colonized by molds depending on the variant (Table 6). The initial time after materials’ moistening is a critical factor for mold development. The first colonizers were *Rhizopus* and *Trichoderma* (Figure 5). These fungi appeared on specimens already in the initial test days and continued to grow until the end of the test. Later, after 12 days’ exposure, the presence of *Achremonium* was observed.

Both hemp and sawdust substrates were attractive for mold growth with the exception of sawdust III where fungal growth was slightly limited and contaminated by one fungal genera *Rhizopus*. This can be explained by the chemical composition of the BS. Besides, the absence of WB in the S III variant is another mold-growth restricting factor. The BB additive to the S II and H II variant did not ensure a higher protective effect against mold although bark contain biologically active substances [29] that could be toxic to microscopic fungi.

A high moisture saturation of MB specimens after water absorption (Figure 3) proved to be the main source of concern regarding mold growth. This experiment approved the importance to avoid an extra moistening of MB materials, especially if they are considered for building insulation. Unpredicted wetting of buildings in case of flood or failure with water leaking, etc., is a serious risk for mold contamination. This aspect has to be foreseen at the planning stage to develop the MB for building industry. However, the existing wood- and non-wood-based composites also encounter mold contamination risk at elevated moisture content. For example, the research on hemp composites with mineral binder [43] and wood composites with synthetic binders [44] also showed limited resistance to mold contamination.

### 3.7. Biodegradability

The adapted EN 14045:2003 standard method was applied to evaluate the disintegration of MB specimens under defined composting conditions. During the test, the temperature in the compost substrate varied between 22 and 24 °C, and the substrate moisture content varied between 70 and 77%. The biodegradation test showed complete disintegration of all MB specimens after 12 weeks, with the exception of two specimens from the sawdust S II variant. The residual parts of these specimens comprised 0.92–1.95% of initial mass that was considered as negligible remains. After a short additional degradation time, a total disintegration of these specimens would most likely occur. The sawdust S II with bark additive was obviously a less susceptible nutrition source for compost microbiota.

Lignocellulose-containing BS and HS were efficiently biodegradable substrates via composting. Cellulose is described as the main source of energy to drive the biological transformations and the consequent temperature rise and chemical changes that are associated with composting. Lignin is the main starting material for the formation of humus, the recalcitrant organic matter that provides the water-holding, ion exchange, and bulking capabilities, which can contribute greatly to soil health and productivity [45]. The fungal mycelium as a part of MB structure served as a protein and nitrogen source for soil microorganisms. Because the reaction proceeds through biochemical pathways, proteins and enzymes are essential, and there has to be a sufficient amount of nitrogen present in the mixture relative to the amount of carbon [45]. As the compost substrate comprises many naturally occurring saprotrophic microorganisms, it provided necessary evidence for the ability of MB specimens to be disintegrated by soil microbiota.

## 4. Conclusions

Development of MB caused changes in mycelium: substrate proportion. Taking into account some impurities in both separated fractions, in general, mycelial biomass in hemp MB increased ~100% (with bran additive) from initial inoculum, with a final fungus: substrate ratio 0.8: 1. Sawdust MB showed ~50% mycelial increment (with bran) and fungus: substrate ratio 0.2–0.3:1. This finding was supported by elemental analyses. Mycelial growth during MB development caused degradation of lignocellulose substrates: up to 7% and 28% of cellulose degradation in sawdust and hemp substrates, respectively, and lignin degradation in the range of 13% from initial in both substrates. The density of sawdust MB was twice as high as that of hemp samples and was affected by the dominant smallest particle fraction in sawdust samples. A larger granulometric fraction in hemp MB ensured higher strength and weakened water absorption performance. WB supplement positively affected fungal growth, resulting in higher biomass yield. Water sorption performance in bran containing sawdust and hemp samples was elevated. Bran containing sawdust MB had improved compressive strength contrary to the hemp samples. BB additive had a positive effect on cellulose and lignin degradation, reducing water absorption by more than 100% and increasing density in sawdust and hemp substrates. The elevated compressive strength was observed in the hemp/bark rather than in the sawdust/bark combination. Perspective combinations regarding mechanical strength performance were hemp/bark and pure hemp MB, as well as the sawdust/bran combinations that were competitive with some commercial EPS types. All MB samples were susceptible to mold contamination after full water immersion, and were similar to other natural fibers containing biomaterials. This indicates the need to avoid MB wetting or to acquire protection of the material with hydrophobic or antifungal substances. The MB exhibited a high biodegradation capability due to their natural composition of hemp, sawdust, bark, bran and fungal mycelium. Thus, MB can compete with the expanded polystyrene materials as an ecological packaging alternative. A high liquid absorption ability of MB has potential applications in the biosorption area.

## Figures and Tables

**Table 1:** The particle size and weight percentage of each fraction in the hemp and sawdust substrates.

Fraction Size mm	Sawdust wt %	Hemp wt %
≥10	5	8
7	1	10
5	4	28
3	21	37
<3	69	17

**Table 2:** Initial and final mycelium biomass by dry weight (%) in mycelium biocomposites (MB). S = sawdust; H = hemp; BB = birch bark; WB = wheat bran. I, II, III = variants.

MB Variant	Initial Mycelium %	Final Mycelium %	Initial Fungus:Substrate Ratio	Final Fungus:Substrate Ratio
S I (WB, water)	0.44	23.9	0.004:1	0.30:1
S II (WB, BB, water)	0.34	16.9	0.003:1	0.20:1
S III (water)	0.44	4.2	0.004:1	0.04:1
H I (WB, water)	0.44	43.7	0.004:1	0.80:1
H II (WB, BB, water)	0.34	44	0.003:1	0.80:1
H III (water)	0.44	42.9	0.004:1	0.75:1

**Table 3:** Chemical composition of mycelium biocomposites’ ingredients. S = birch sawdust; H = hemp shives; BB = birch bark; WB = wheat bran.

Ingredient	Cellulose %	Klason Lignin %	Ash %	Other Components ** %
S	48.33 ± 0.20	23.39 ± 0.66	0.42 ± 0.02	27.86
H	44.95 ± 0.58	24.30 ± 0.07	1.99 ± 0.02	28.76
BB	15.60 ± 0.90	79.00 ± 9.50 *	0.65 ± 0.12	4.73
WB	12.60 ± 0.40	10.28 ± 0.12	5.62 ± 0.02	71.5

* Phenolics (lignin, suberin, extractives), ** Mainly hemicelluloses.

**Table 4:** Ash content and elemental composition N, C, H at three mycelium biocomposites (MB) development stages: start point (0 days), 14 days, and 21 days (final sample). S = sawdust; H = hemp; I, II, III = variants.

MB Variant	Ash %	Nitrogen %	Carbon %	Hydrogen %
S I-0	0.75 ± 0.02	0.26 ± 0.02	47.47 ± 0.15	5.98 ± 0.15
S II-0	1.10 ± 0.12	0.34 ± 0.07	52.31 ± 4.12	6.41 ± 0.42
S III-0	0.42 ± 0.02	0.25 ± 0.09	47.48 ± 0.08	5.89 ± 0.10
S I-14	1.35 ± 0.02	0.94 ± 0.13	46.66 ± 0.47	6.03 ± 0.17
S II-14	1.67 ± 0.05	0.95 ± 0.01	51.72 ± 1.87	6.44 ± 0.03
S III-14	0.57 ± 0.07	0.23 ± 0.04	46.67 ± 0.12	5.92 ± 0.09
S I-21	1.64 ± 0.01	0.77 ± 0.06	46.83 ± 0.08	6.00 ± 0.13
S II-21	1.94 ± 0.01	0.79 ± 0.07	56.76 ± 0.95	6.83 ± 0.18
S III-21	0.65 ± 0.00	0.31 ± 0.05	47.25 ± 0.17	6.15 ± 0.05
H I-0	2.53 ± 0.08	0.71 ± 0.05	46.92 ± 0.35	5.93 ± 0.04
H II-0	1.70 ± 0.04	0.73 ± 0.08	51.91 ± 0.92	6.44 ± 0.09
H III-0	1.99 ± 0.02	0.60 ± 0.04	46.93 ± 0.22	5.98 ± 0.10
H I-14	2.67 ± 0.02	1.21 ± 0.05	46.30 ± 0.22	5.98 ± 0.05
H II-14	1.98 ± 0.04	1.04 ± 0.06	47.10 ± 0.55	5.93 ± 0.15
H III-14	2.41 ± 0.02	1.09 ± 0.11	45.69 ± 1.18	5.75 ± 0.10
H I-21	4.40 ± 0.03	1.24 ± 0.13	45.84 ± 0.32	5.89 ± 0.21
H II-21	3.72 ± 0.00	0.94 ± 0.06	54.74 ± 1.96	6.32 ± 0.43
H III-21	3.89 ± 0.06	1.08 ± 0.09	46.56 ± 0.27	5.89 ± 0.04

**Table 5:** Compressive properties of mycelium biocomposites (MB) after 21 day development.

MB Variant	Density g cm^−3^	σ_10_ MPa	E MPa
S I-21	0.200 ± 0.030	0.225 ± 0.028	2.910 ± 0.465
S II-21	0.215 ± 0.021	0.178 ± 0.053	2.202 ± 0.841
S III-21	0.184 ± 0.032	0.029 ± 0.009	0.296 ± 0.102
H I-21	0.103 ± 0.003	0.162 ± 0.020	2.412 ± 0.587
H II-21	0.134 ± 0.011	0.198 ± 0.008	2.913 ± 0.210
H III-21	0.105 ± 0.009	0.190 ± 0.015	2.938 ± 0.396

**Table 6:** Mold growth on mycelium biocomposites (MB) during 14 days exposure. S = sawdust; H = hemp; I, II, III = variants.

MB Variant	Time (Days)/Evaluation Grade (1–4)					Molds Identified
	0	3	7	12	14	
S I	0	4	4	4	4	*Rhizopus*, *Trichoderma*, *Achremonium*
S II	0	4	4	4	4	*Rhizopus*, *Trichoderma*, *Achremonium*
S III	0	3	3	3	3	*Rhizopus*
H I	0	4	4	4	4	*Rhizopus*, *Trichoderma*, *Achremonium*
H II	0	4	4	4	4	*Rhizopus*, *Trichoderma*, *Achremonium*
H III	0	4	4	4	4	*Rhizopus*, *Trichoderma*, *Achremonium*

## Data Availability

Data will be available upon reasonable request.
